# Primary intraosseous osteolytic meningioma: a case report and review of the literature

**DOI:** 10.1186/s12883-019-1392-5

**Published:** 2019-07-23

**Authors:** Sae Min Kwon, Yong Ko, Seong Sik Bang

**Affiliations:** 1 Departments of Neurosurgery, College of Medicine, Hanyang University, 17 Haengdang-dong, Seongdong-gu, 133-792 Seoul, Republic of Korea; 2 Department of Neurosurgery, Keimyung University School of Medicine, 1095 Dalgubeol-daero, Dalseo-gu, Daegu, 42601 Republic of Korea; 3 Departments of Pathology, College of Medicine, Hanyang University, 17 Haengdang-dong, Seongdong-gu, 133-792 Seoul, Republic of Korea

**Keywords:** Intraosseous, Meningioma, Osteolysis

## Abstract

**Background:**

Primary intraosseous meningioma is a subset of extradural meningioma that arises in the bone, and only a few cases have been reported to date.

**Case presentation:**

An 80-year-old man presented with decreased hearing on the right side accompanied by a disturbance of balance 10 months prior to admission. Magnetic resonance imaging revealed an 8 × 7 cm osteolytic mass in the right posterior fossa related to the petrous bone, with extension to the cervical region. During surgery, the tumor was found to be located extradurally, with no invasion of the dura. The tumor was removed entirely, apart from a small portion around the jugular foramen to avoid lower cranial nerve injury.

**Conclusion:**

The final diagnosis was primary intraosseous osteolytic meningioma with atypical pathology. Here, we report a rare case of an osteolytic skull lesion in the skull base not invading the dura and with extensive bone destruction.

**Electronic supplementary material:**

The online version of this article (10.1186/s12883-019-1392-5) contains supplementary material, which is available to authorized users.

## Background

Meningiomas are common intradural lesions that arise from the arachnoid cap cells of the arachnoid layer. In contrast, primary extradural meningioma is a relatively rare entity, accounting for less than 2% of all meningiomas [1, 2]. They may arise from other locations, such as the skin, orbit, nasopharynx, and neck [3–5]. Primary intraosseous meningioma, which arises in the bone, is a subset of primary extradural meningioma, and only a few cases have been reported [1, 3]. Here, we report a recent case of primary intraosseous osteolytic meningioma with extension to the cervical region which was successfully removed.

## Case presentation

An 80-year-old man presented with a progressive decrease in hearing on the right side accompanied by dizziness and disturbance of balance 10 months prior to admission. The neurological examination revealed right hypoglossal nerve palsy. Audiometry documented complete sensorineural hearing loss on the right side.

**Fig. 1:**
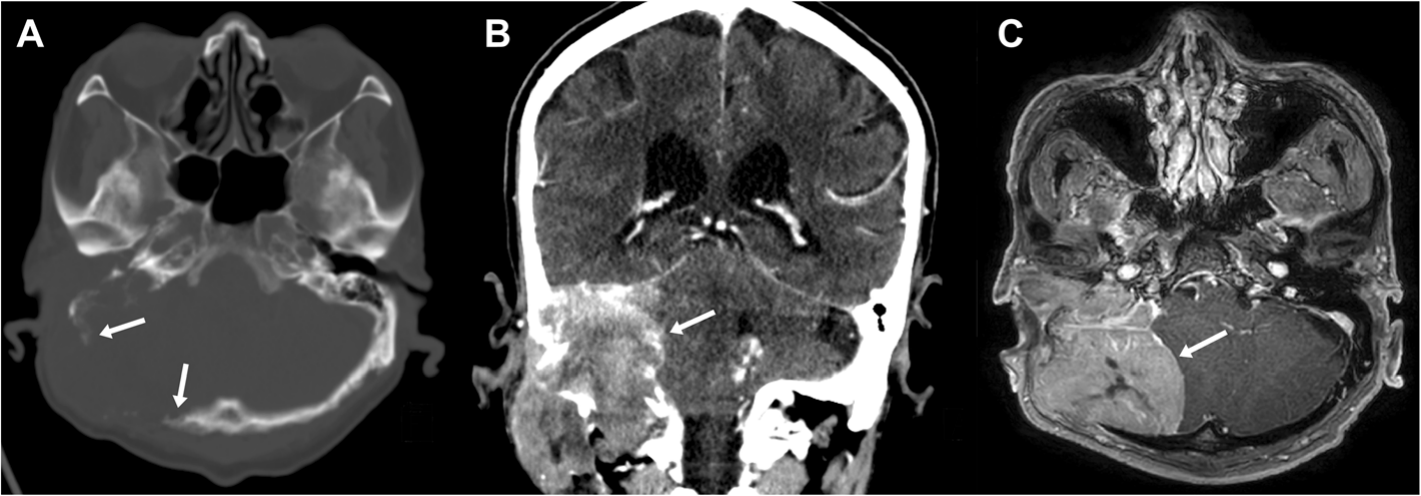
Preoperative imaging. Axial computed tomography (CT) scan with bone window **(a**) shows a destructive osteolytic mass lesion in the right temporal-posterior fossa region. Coronal CT scan (**b**) and magnetic resonance imaging (**c**) revealed an 8 × 7 cm homogenous mass extending to the cervical region

Skull x-ray and cranial computed tomography (CT) scans showed a large osteolytic lesion with bone destruction, including the temporal bone, occipital bone, clivus, jugular foramen, and hypoglossal canal (Fig. 1a and b). Magnetic resonance imaging (MRI) revealed an 8 × 7 cm homogeneous enhancing mass in the right posterior fossa related to the petrous part of the temporal bone, with extension to the cervical region (Fig. 1c). The cerebellum was displaced, and definite brain invasion was not seen. The preoperative diagnosis was a temporal bone origin malignancy such as squamous cell carcinoma or meningioma with invasion of the petrous bone.

**Fig. 2:**
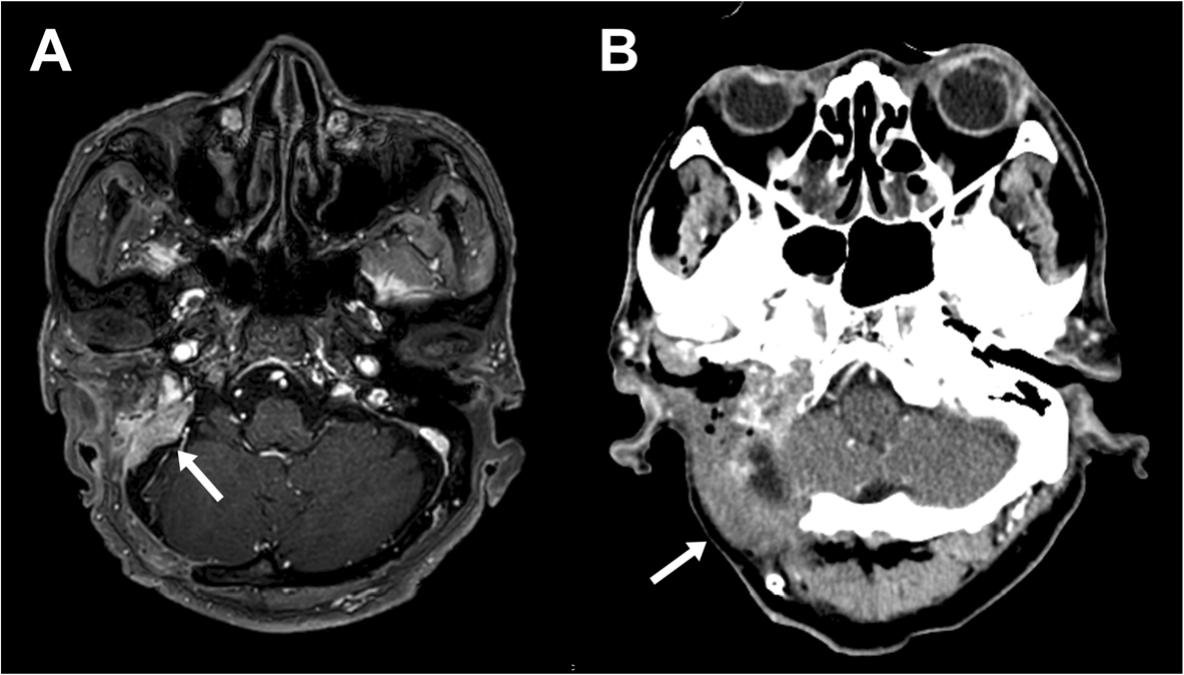
Postoperative magnetic resonance imaging (**a**) shows a small residual tumor around the jugular foramen, and computed tomography scan (**b**) demonstrates the sternocleidomastoid muscle which fills the tumor removal space

The patient underwent surgery to obtain a pathological diagnosis and for complete removal of the mass. A C-shaped postauricular skin incision was made that extended to the neck. The scalp was reflected anteriorly, and the mass infiltrating the subcutaneous tissue was exposed. The lesion appeared as a firm gray mass that had destroyed the temporal and occipital bones. The dura was intact with no invasion, and the lesion was easily peeled off. For the cervical part of the tumor, the major vessels were secured inferiorly, and the mass was removed up to the skull base. The tumor was removed entirely, except for a small portion around the jugular foramen to avoid lower cranial nerve injury. Finally, the large empty space was filled with a sternocleidomastoid muscle flap (Fig. 2). There were no neurological deficits after surgery.

**Fig. 3:**
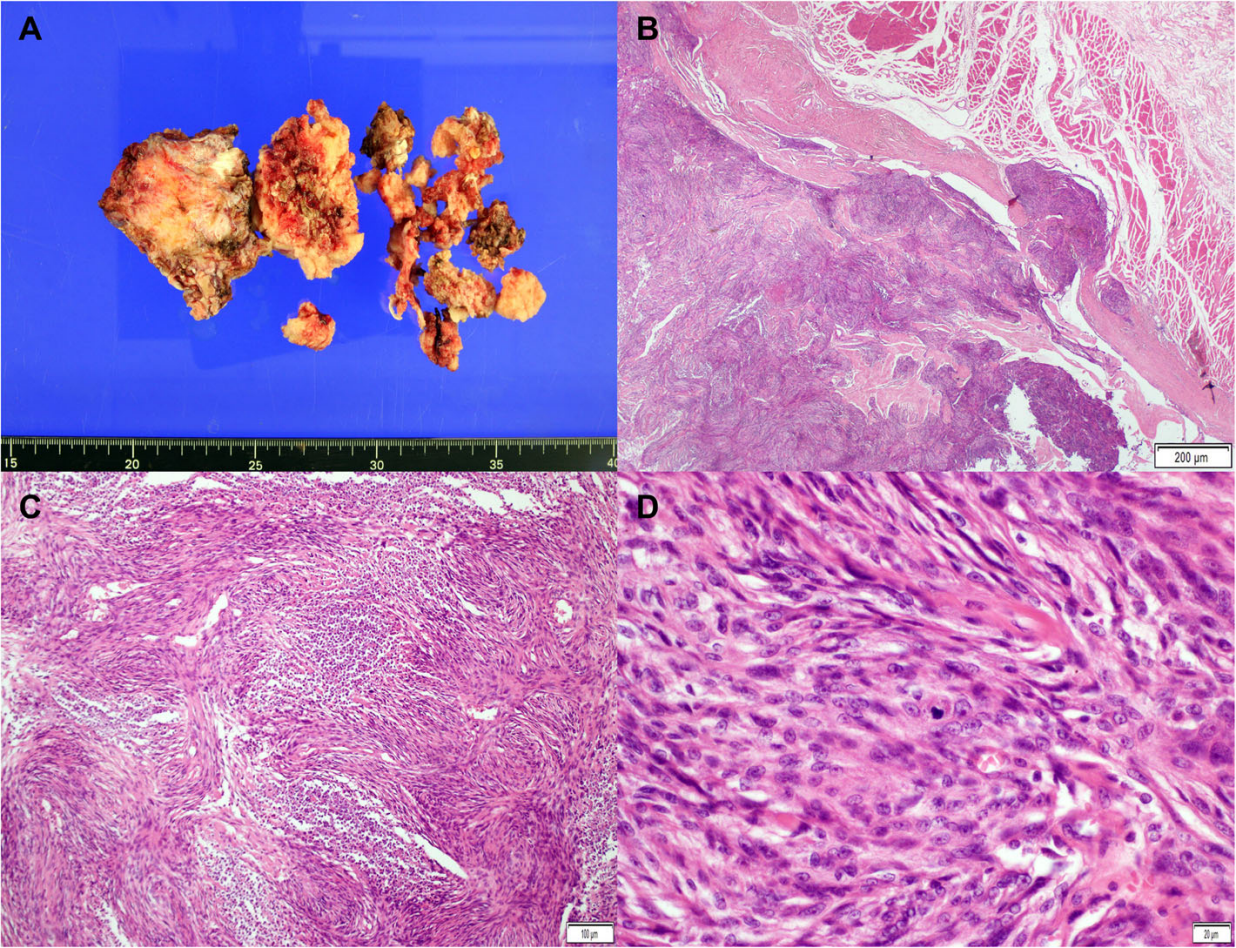
Histopathologic findings of atypical meningioma. The fragmented specimen (**a**) is seen as grayish-white solid masses. On microscopic examination, the tumor infiltrated the adjacent soft tissue (**b**, hematoxylin and eosin [H&E] stain, × 20, scale bar = 200 μm) and showed a whorled appearance and multifocal necrosis (**c**, H&E stain, × 100, scale bar = 100 μm). The tumor cells are composed of spindle cells with prominent nucleoli and ill-defined cytoplasm. Many mitoses are visible (**d**, H&E stain, × 400, scale bar = 20 μm)

Histopathological studies confirmed a WHO (World Health Organization) grade II atypical meningioma with up to 6 mitoses per 10 high-power fields (Fig. 3). The Ki-67 proliferation index was 15%. The results of immunohistochemical staining are provided in Additional file 1: Figure S1.

## Discussion and conclusions

Primary intraosseous meningiomas are rare cranial lesions that arise from the bone, and they represent the most common type of primary extradural meningiomas [1–3]. The majority of intraosseous meningiomas are osteoblastic and cause hyperostosis, which may mimic fibrous dysplasia. In contrast, and more rarely, they may present as an osteolytic skull lesion [6, 7]. Reportedly, less than 20% of intraosseous meningiomas are osteolytic [8].

Primary extradural meningiomas are most commonly found in skull convexities, the paranasal sinus, and the middle ear but rarely in the skull base. Liu et al. reported 170 cases of extradural meningiomas in the head, and only 5.8% were located in the skull base [9]. Notably, there are few reports of osteolytic intraosseous meningiomas in the skull base. To date, 50 cases of osteolytic subtypes (including the present case) have been reported in the English literature (Table 1). Of these, only seven were located in the skull base, and all but two originated from the petrous bone.

**Table 1:** Reports of primary intraosseous osteolytic meningiomas

Reference, year	Sex/age	Location	Type	Pathology
Klein et al., 1975 [10]	F/66	Parietal	IIIC	Meningothelial
McWhorter et al., 1976 [11]	M/42	Temporal	IIC	Benign
Palma et al., 1977 [12]	M/18	Frontal	IIIC	Fibroblastic
Pearl et al., 1979 [13]	F/44	Frontal	IIC	Meningothelial
Ohaegbulam et al., 1979 [14]	M/31	Frontal	IIIC	Fibroblastic
Young et al., 1983 [15]	M/71	Frontal	IIC	Benign
Kaneko et al., 1988 [16]	F/71	Frontoparietal	IIIC	Fibroblastic
Lee et al., 1988 [17]	F/71	Frontoparietal	IIC	Malignant
Oka et al., 1989 [18]	F/79	Frontoparietal	IIIC	Transitional
Ammirati et al., 1990 [19]	M/21	Petrous	IIIB	Benign
Kulali et al., 1991 [20]	M/50	Occipital	IIIC	Transitional
Ito et al., 1992 [21]	F/72	Frontoparietal	IIC	Meningothelial
Fujita et al., 1993 [22]	M/42	Petrous	IIIB	Malignant
Ghobashy and Tobler, 1994 [23]	F/65	Frontal	IIC	Transitional
Parington et al., 1995 [24]	F/84	Frontotemporal	IIIC	Atypical
Levin et al., 1995 [25]	N/A	Calvaria	N/A	N/A
Kuzeyli et al., 1996 [26]	M/6	Temporal	IIC	Meningothelial
Changhong et al., 1997 [27]	F/42	Occipital	IIC	Malignant
Muthukumar et al., 1997 [28]	M/55	Parietal	IIIC	Meningothelial
	M/50	Temporoparietal	IIC	Meningothelial
	M/60	Frontal	IIIC	Meningothelial
Kudo et al., 1998 [29]	F/56	Parietooccipital	IIIC	Meningothelial
Okamoto et al., 2000 [30]	F/78	Parietal	IIC	Microcystic
Yamazaki et al., 2001 [31]	M/62	Occipital	IIIB	Meningothelial
Rosahl et al., 2004 [32]	M/38	Petrous	IIB	Meningothelial
Tokgoz et al., 2005 [33]	M/44	Frontoparietal	IIIC	Chordoid
Bassiouni et al., 2006 [34]	M/47	Parietal	IIIC	Benign
	F/46	Temporal	IIC	Meningothelial
	F/57	Parietal	IIC	Fibroblastic
	F/62	Frontal	IIC	Atypical
	M/34	temporal	IIIC	Meningothelial
Al-Khawaja et al., 2007 [35]	M/50	Parietal	IIC	Meningothelial
Sheikhrezaie et al., 2009 [36]	M/62	Frontoparietal	IIIC	Benign
Yener et al., 2009 [37]	M/78	Parietal	IIC	Meningothelial
Hong et al., 2010 [38]	M/52	Parietal	N/A	Benign
	M/73	Occipital	N/A	Anaplastic
Kim et al., 2012 [39]	M/68	Parietal	IIIC	Atypical
	F/74	Frontal	IIIC	Papillary
Akhaddar and Ennouali, 2014 [40]	F/37	Frontal	IIC	Meningothelial
Tang et al., 2014 [41]	F/82	Parietal	IIC	Meningothelial
Yun and Lee, 2014 [42]	F/65	Frontal	IIIC	Atypical
Kim et al., 2014 [43]	F/44	Sphenoid	IIIB	Transitional
Bujok and Bienioszek, 2014 [44]	F/59	Frontal	IIC	Microcystic
Kwon et al., 2015 [45]	M/69	Parietal	IIIC	Meningothelial
Hong et al., 2015 [46]	M/61	Frontoparietal	IIC	Benign
Ben Nsir et al., 2016 [47]	M/42	Petrous	IIIB	Clear cell
Bohara et al., 2016 [48]	M/38	Parietal	IIIC	Atypical
Mouri et al., 2017 [49]	F/76	Frontal	IIIC	Transitional
Richardson et al., 2017 [50]	M/23	Frontal	IIC	Benign
Present case	M/80	Petrous	IIIB	Atypical

The exact origin of extradural meningiomas is unclear, but several theories have been proposed. Their unusual locations are assumed to be the result of the aberrant differentiation or misplacement of undifferentiated mesenchymal stem cells [51]. Alternatively, extradural meningiomas may arise from differentiated arachnoid cap cells associated with blood vessels or nerves traversing the skull [52, 53]. Another theory proposes that they originate from arachnoid cap cells that get trapped in the cranial sutures during embryogenesis or molding of the cranium at birth [20, 21, 54]. Trauma with skull fracture has also been proposed as a causative factor of some extradural meningiomas, suggesting direct dural entrapment within bone fragments at the time of trauma [55].

The osteolytic subtype of intraosseous meningiomas is often misdiagnosed as a primary or secondary bone tumor due to its radiological appearance. The differential diagnosis of a solitary osteolytic skull lesion includes hemangioma, chondroma, chondrosarcoma, eosinophilic granuloma, epidermoid cyst, giant cell tumor, myeloma, and metastatic skull tumor [6, 23, 33].

Primary extradural meningiomas were practically classified according to their location by Lang and colleagues (Table 2) [3]. Therefore, intraosseous meningiomas could be considered Type II or Type III extradural meningiomas. Based on this classification, the present case falls into the type IIIB category due to the presence of extracalvarial extension. This classification is helpful in predicting the risk of tumor recurrence. The IIC and IIIC subtypes rarely recur after complete resection, whereas the IIB and IIIB subtypes have a reported lifetime risk of recurrence of 26% [3].

**Table 2:** Primary extradural meningioma classification by Lang et al. 2000 [3]

Type	Description	Subtype
I	Purely extracalvarial with no bony attachment	
II	Purely calvarial	B (skull base)
		C (convexity)
III	Calvarial with extracalvarial extension	B (skull base)
		C (convexity)

Histopathological features are also important factors affecting tumor recurrence and prognosis. Recurrence was noted in 22% of benign intraosseous meningiomas in the literature, while it was reportedly 33% in cases of tumors with atypical or malignant pathology. In addition, aggressive atypical or malignant meningiomas had a significantly higher mortality of 29% compared to tumors with benign features (4.8%) [3]. Osteolytic meningiomas may have a higher incidence of atypical or malignant features [6]. In previous reports, benign features were reported in 87–89% of all extradural meningiomas, whereas in our literature review of osteolytic intraosseous meningiomas, 26% of cases were WHO grade II or III [3, 8].

Wide surgical excision is the main treatment for extradural meningiomas, and it is potentially curative if complete resection is achieved [6, 7]. In the present case, a small portion of the tumor near the jugular foramen could not be removed due to the possibility of cranial nerve injury. In the case of skull base lesions that cannot be totally resected, decompression of vital neural structures is performed.

In conclusion, we performed surgical treatment for a rare case of primary osteolytic intraosseous meningioma in the skull base with extension to the cervical area. The histopathologic diagnosis was atypical meningioma. If possible, complete resection is the treatment of choice, and serial follow-up should be done to confirm recurrence or progression.

## Additional file

Additional file 1: **Figure S1.** Immunohistochemical staining results. The tumor showed a wild-type p53 pattern (A, × 200) and exhibited strong cytoplasmic expression of β-catenin (B, × 200). Some tumor cells exhibited weak membranous expression of EGFR (C, × 200). The tumor was negative for Bcl-2 (D, × 200). The tumor shows membrane and cytoplasmic immunopositivity for EMA (E, × 200) and negative for S-100 protein (F, × 200). Vimentin is diffusely expressed in the cytoplasm of tumor cells (G, × 200). The Ki-67 proliferation index is estimated to be approximately 15% (H, × 200). Scale bar = 100 μm. (DOCX 8214 kb)

## Data Availability

Not applicable.

## Abbreviations

CT: Computed tomography
MRI: Magnetic resonance imaging
WHO: World Health Organization
