# Supplementary material for: Primary intraosseous osteolytic meningioma: a case report and review of the literature
Source: BMC Neurol. 2019 Jul 23;19:176. doi: 10.1186/s12883-019-1392-5 (PMC6647308; doi:10.1186/s12883-019-1392-5)
Supplement: Supplementary file 1 — Figure S1. Immunohistochemical staining results. The tumor showed a wild-type p53 pattern (A, × 200) and exhibited strong cytoplasmic expression of β-catenin (B, × 200). Some tumor cells exhibited weak membranous expression of EGFR (C, × 200). The tumor was negative for Bcl-2 (D, × 200). The tumor shows membrane and cytoplasmic immunopositivity for EMA (E, × 200) and negative for S-100 protein (F, × 200). Vimentin is diffusely expressed in the cytoplasm of tumor cells (G, × 200). The Ki-67 proliferation index is estimated to be approximately 15% (H, × 200). Scale bar = 100 μm. (DOCX 8214 kb) [file 12883_2019_1392_MOESM1_ESM.docx]

**Additional File 1**


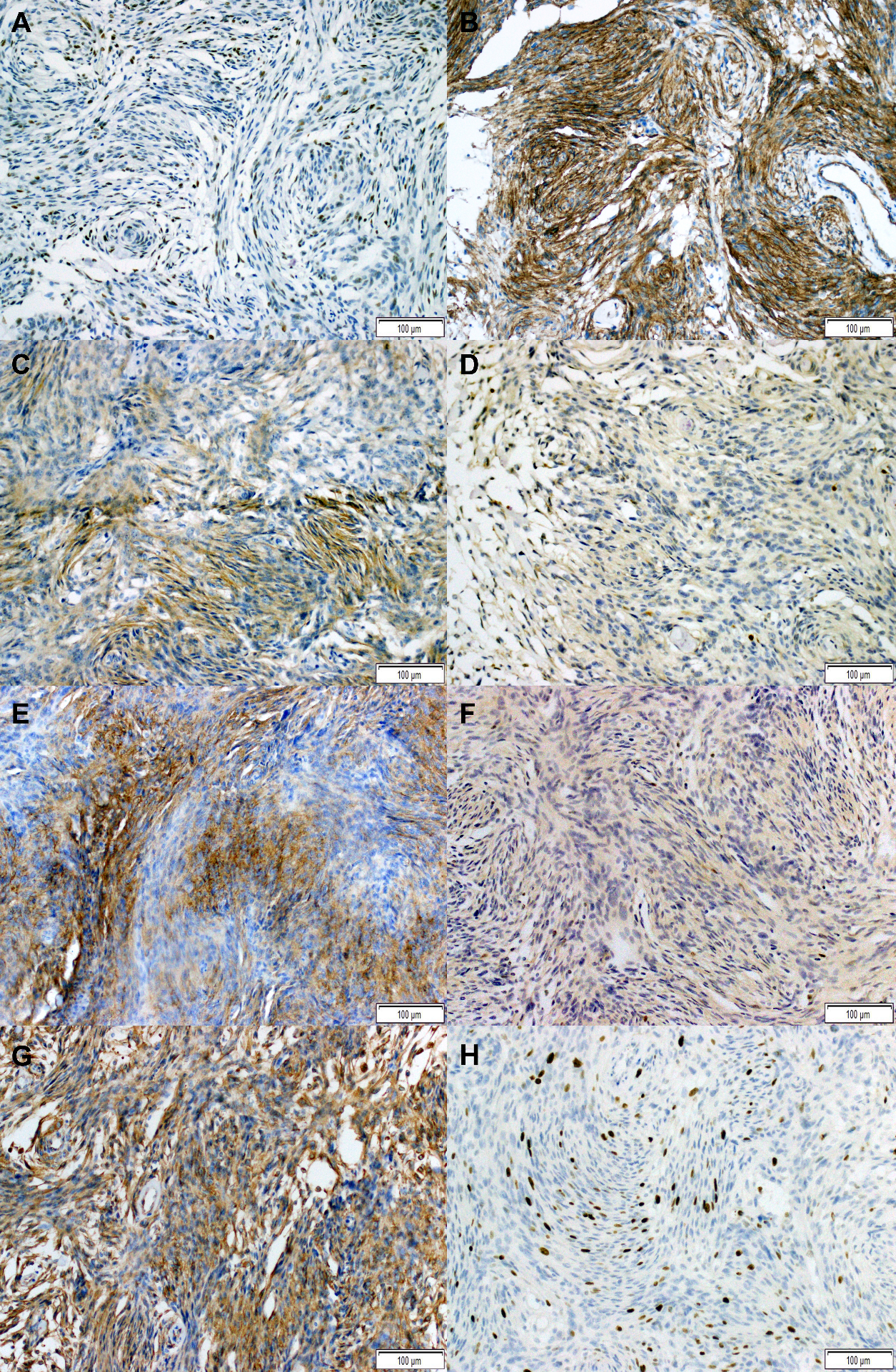


**Additional Figure 1.** Immunohistochemical staining results. The tumor showed a wild-type p53 pattern (A, ×200) and exhibited strong cytoplasmic expression of β-catenin (B, ×200). Some tumor cells exhibited weak membranous expression of EGFR (C, ×200). The tumor was negative for Bcl-2 (D, ×200). The tumor shows membrane and cytoplasmic immunopositivity for EMA (E, x200) and negative for S-100 protein (F, x200). Vimentin is diffusely expressed in the cytoplasm of tumor cells (G, x200). The Ki-67 proliferation index is estimated to be approximately 15% (H, x200). Scale bar = 100 ㎛.
